# Supplementary material for: Silencing LncRNA CASC9 inhibits proliferation and invasion of colorectal cancer cells by MiR-542-3p/ILK
Source: PLoS One. 2022 Apr 15;17(4):e0265901. doi: 10.1371/journal.pone.0265901 (PMC9012350; doi:10.1371/journal.pone.0265901)
Supplement: S1 Raw images — (PDF) [file pone.0265901.s001.pdf]

1. EDU

(1) Fig2D

Hoechst:

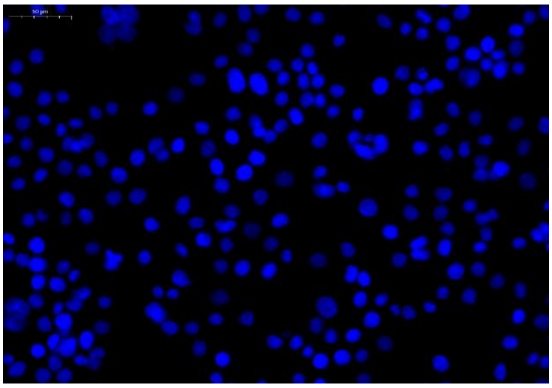

SW620-sh-NC

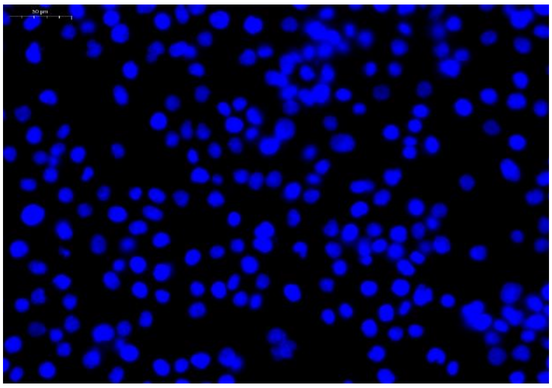

SW620-sh-CASC9-2

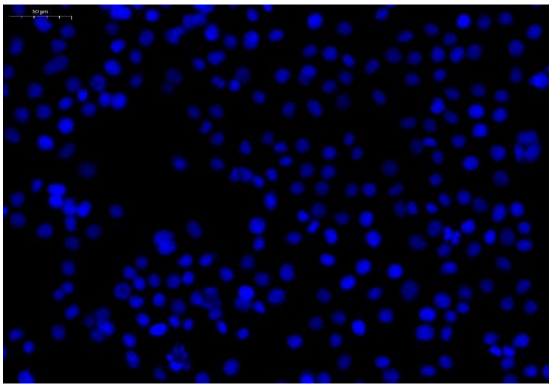

HCT116-sh-NC

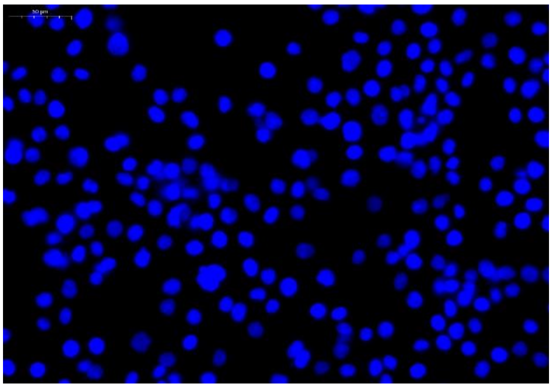

HCT116-sh-CASC9-2

EdU:

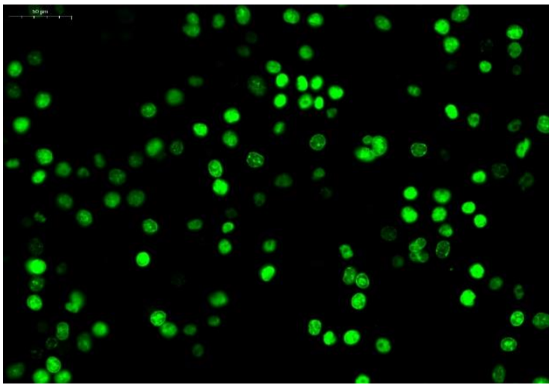

SW620-sh-NC

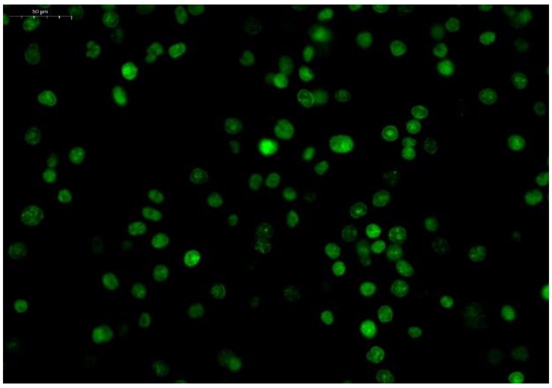

SW620-sh-CASC9-2

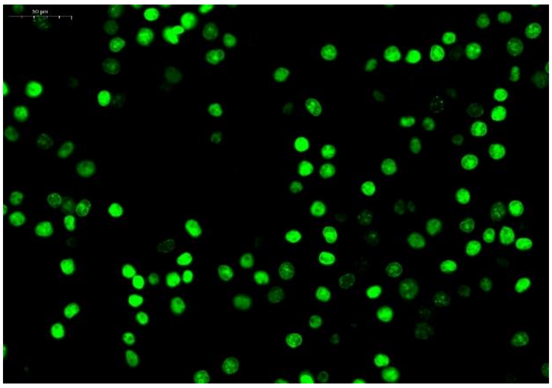

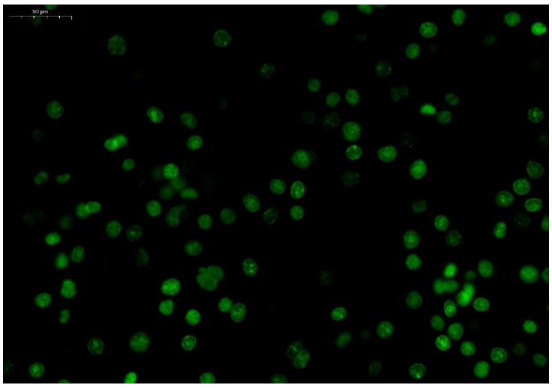

HCT116-sh-NC

HCT116-sh-CASC9-2

Merge:

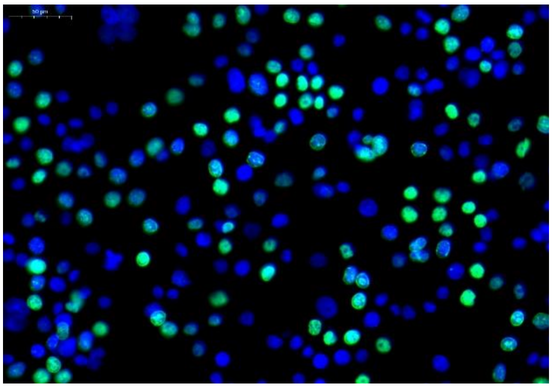

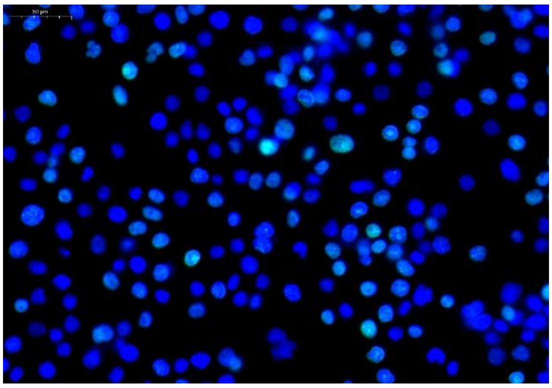

SW620-sh-NC

SW620-sh-CASC9-2

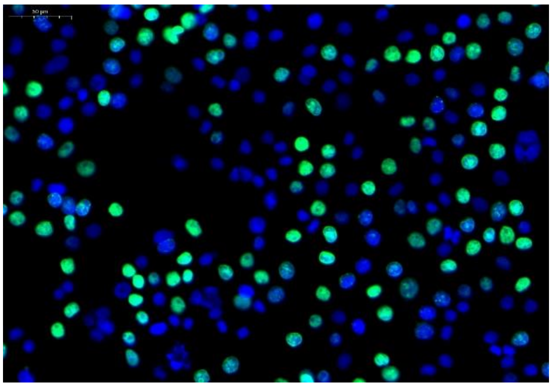

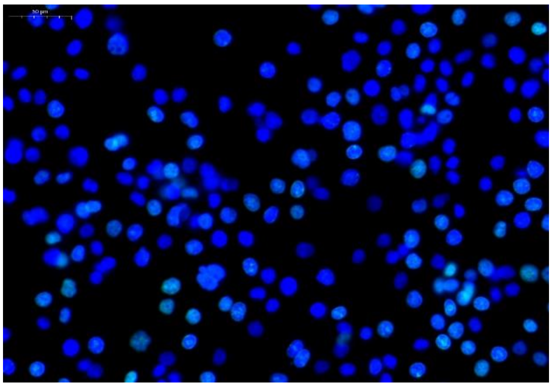

HCT116-sh-NC

HCT116-sh-CASC9-2

(2) Fig5D

Hoechst:

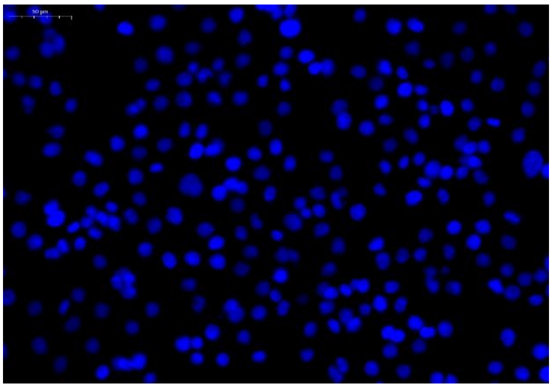

SW620-miR-NC

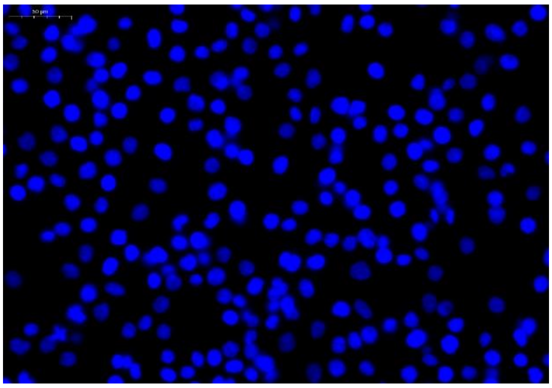

SW620-miR-542-3p mimics

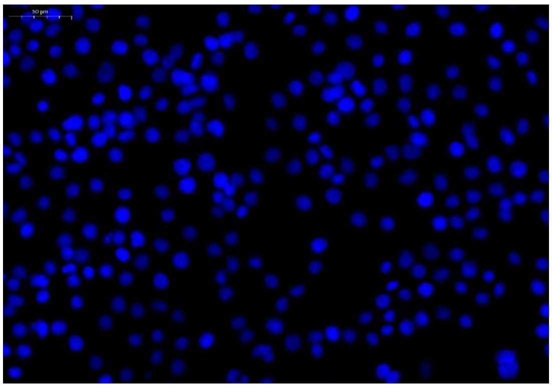

HCT116- miR-NC

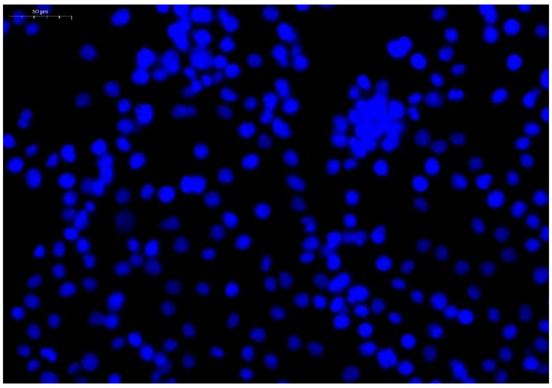

HCT116- miR-542-3p mimics

EdU:

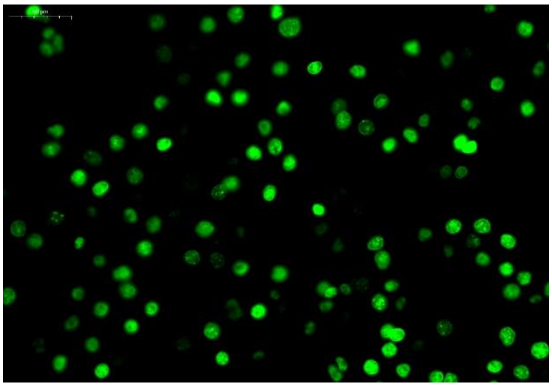

SW620-miR-NC

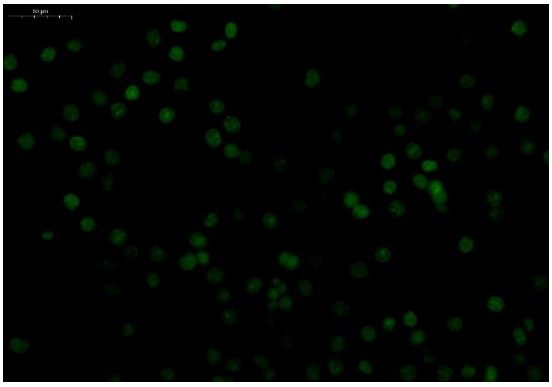

SW620-miR-542-3p mimics

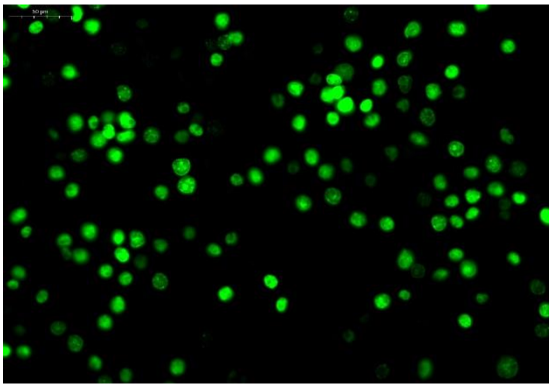

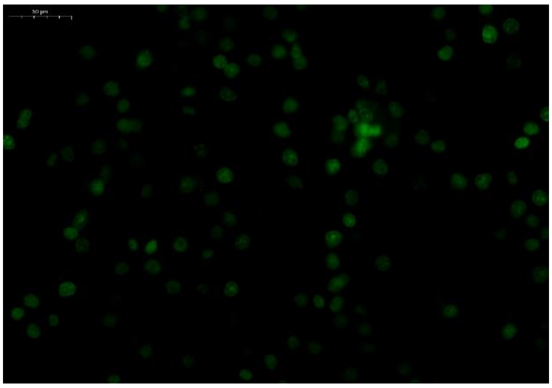

HCT116- miR-NC HCT116- miR-542-3p mimics

Merge:

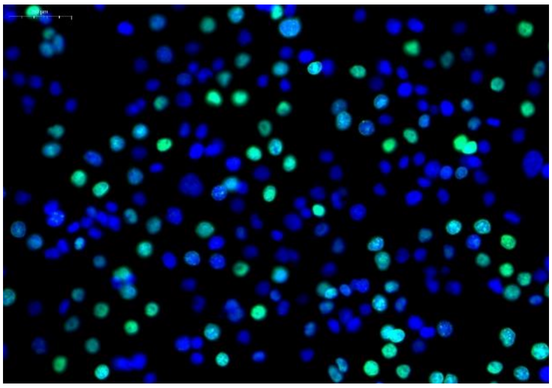

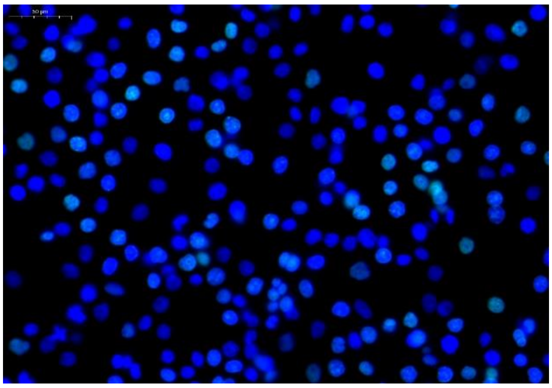

SW620-miR-NC SW620-miR-542-3p mimics

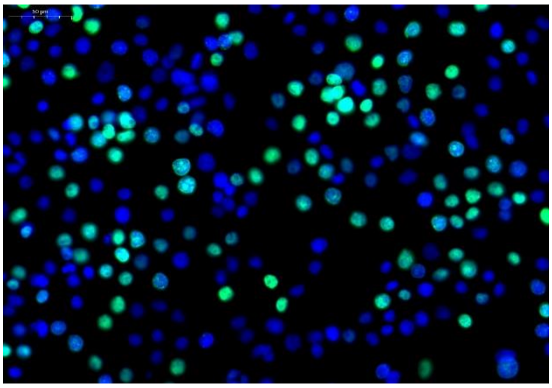

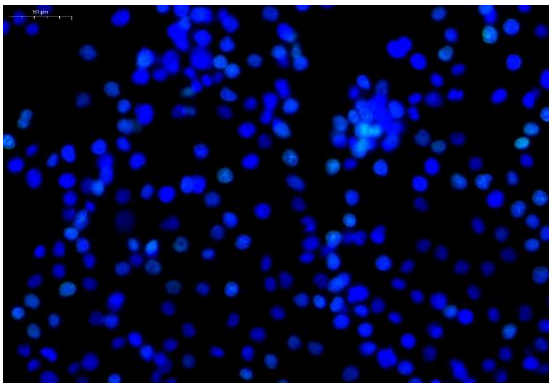

HCT116- miR-NC HCT116- miR-542-3p mimics

(3) Fig7B

SW620:

Hoechst:

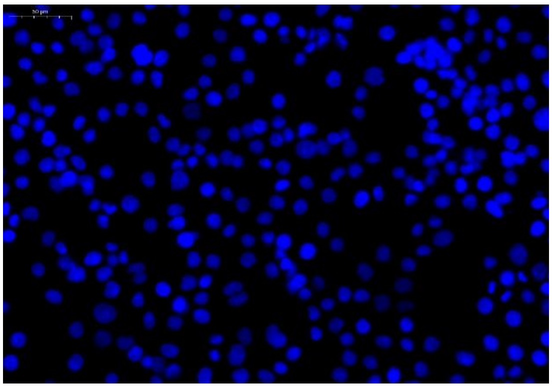

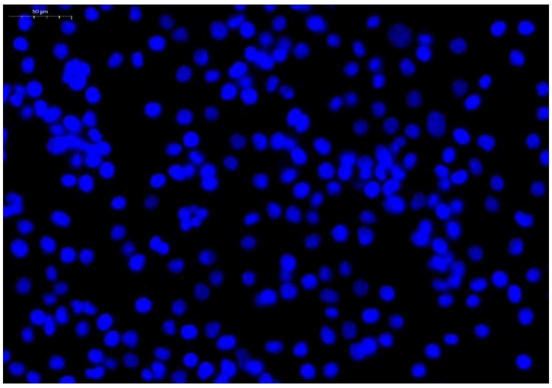

Blank sh-CASC9-2

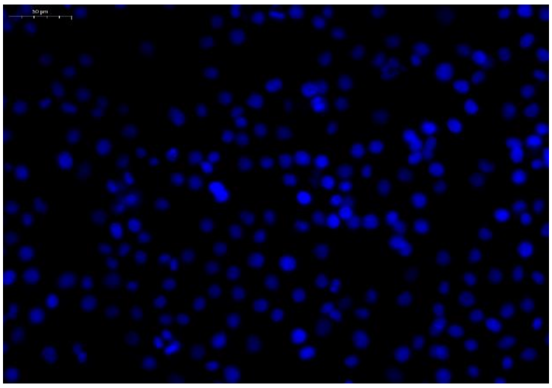

sh-CASC9-2+pcDNA3.1-ILK

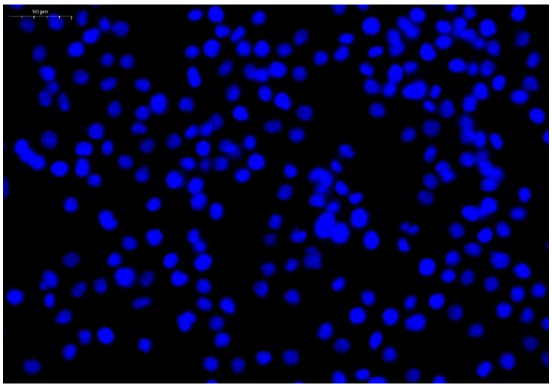

miR-542-3p mimics

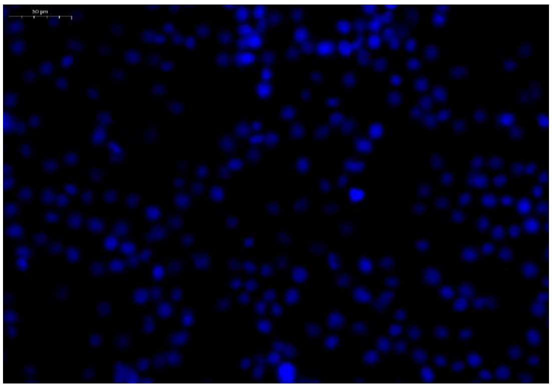

miR-542-3p mimics+ pcDNA3.1-ILK

EdU:

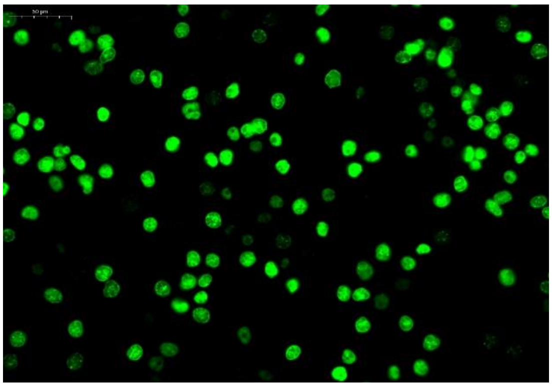

Blank

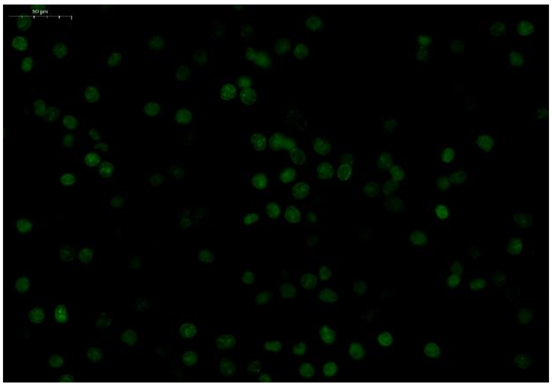

sh-CASC9-2

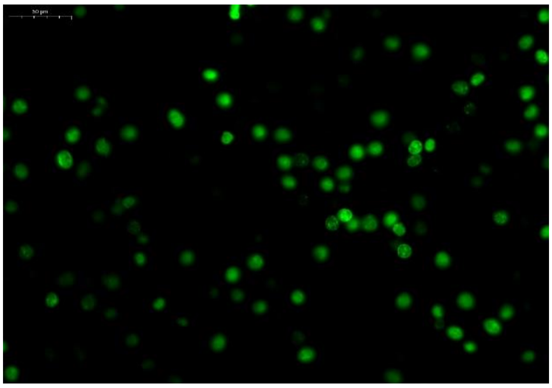

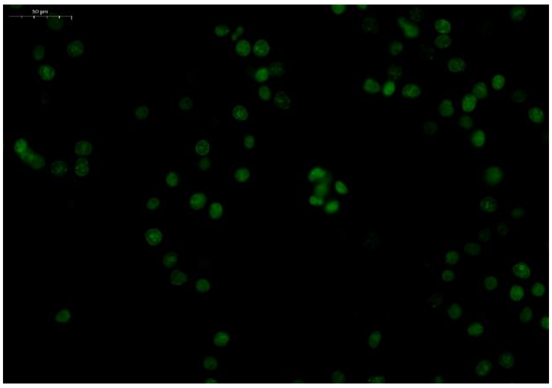

sh-CASC9-2+pcDNA3.1-ILK

miR-542-3p mimics

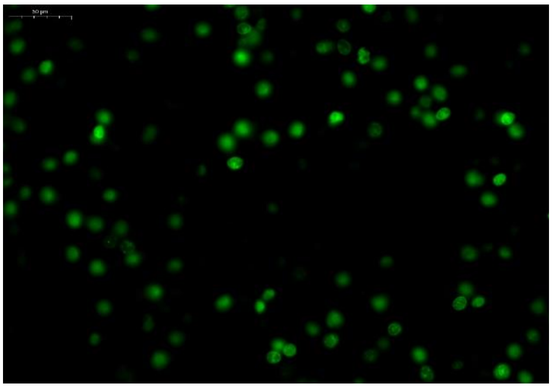

miR-542-3p mimics+ pcDNA3.1-ILK

Merge:

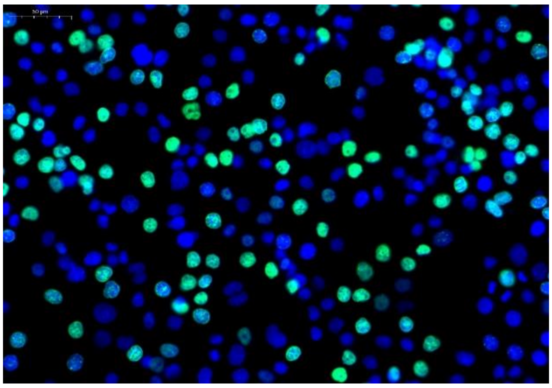

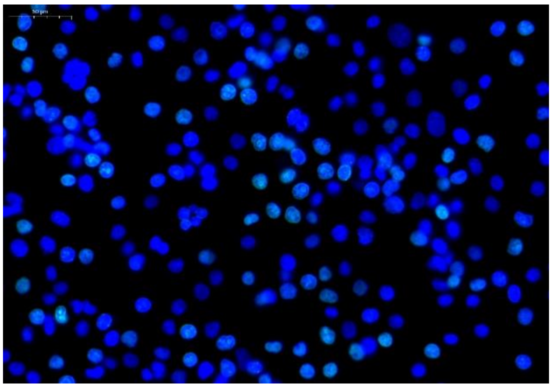

Blank

sh-CASC9-2

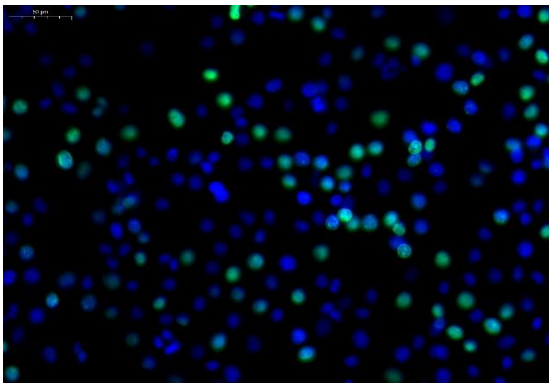

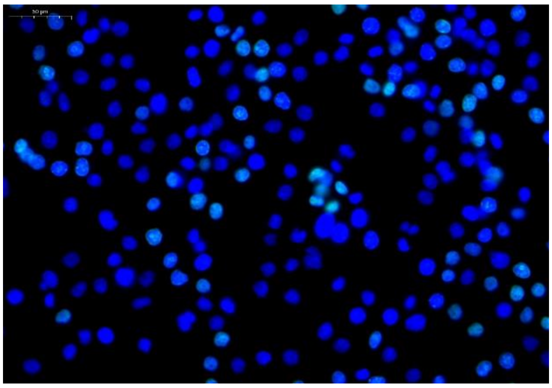

sh-CASC9-2+pcDNA3.1-ILK

miR-542-3p mimics

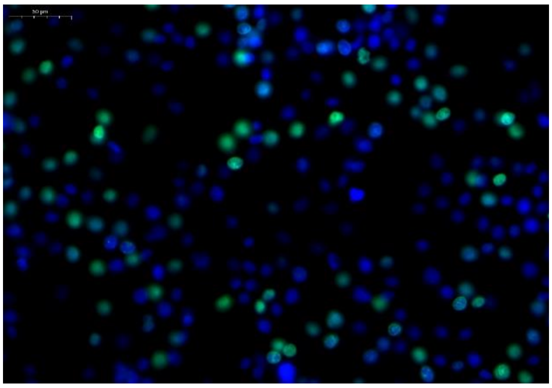

miR-542-3p mimics+ pcDNA3.1-ILK

HCT-116:

Hoechst:

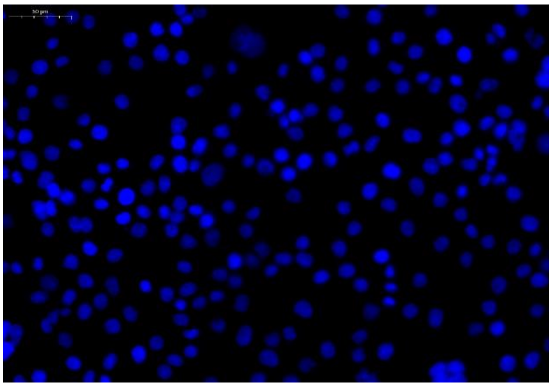

Blank

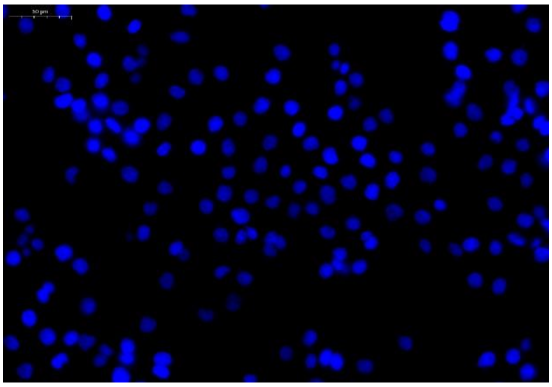

sh-CASC9-2

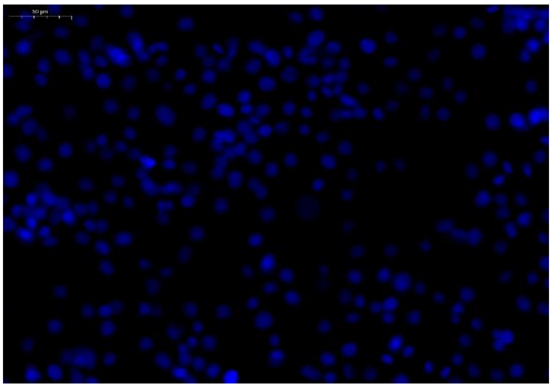

sh-CASC9-2+pcDNA3.1-ILK

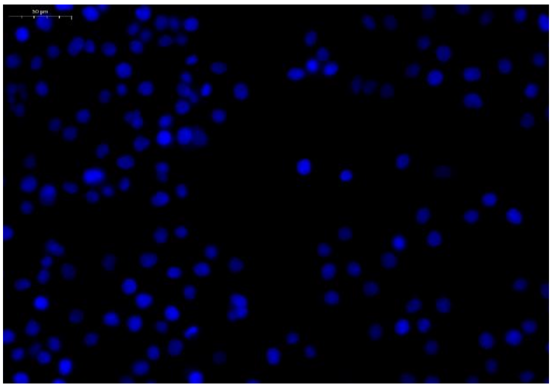

miR-542-3p mimics

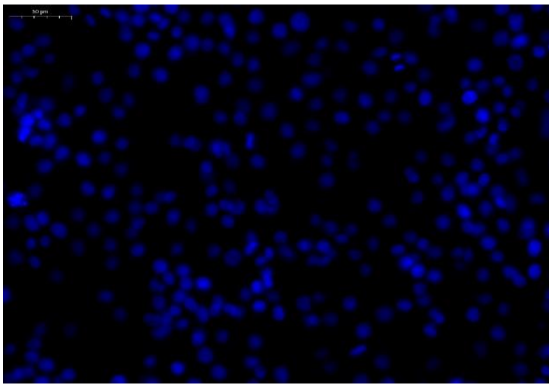

miR-542-3p mimics+ pcDNA3.1-ILK

EdU:

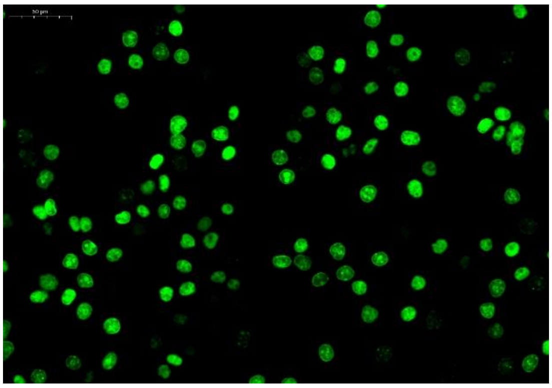

Blank

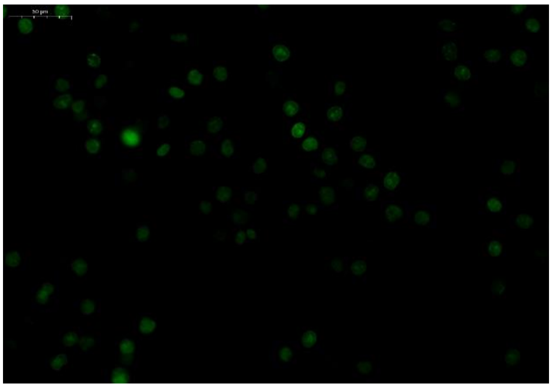

sh-CASC9-2

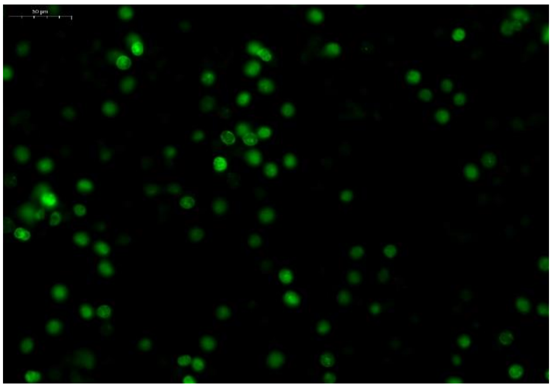

sh-CASC9-2+pcDNA3.1-ILK

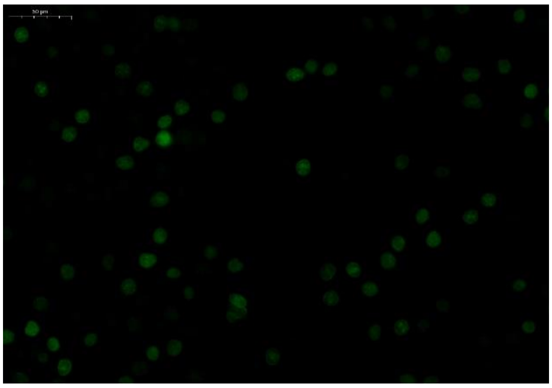

miR-542-3p mimics

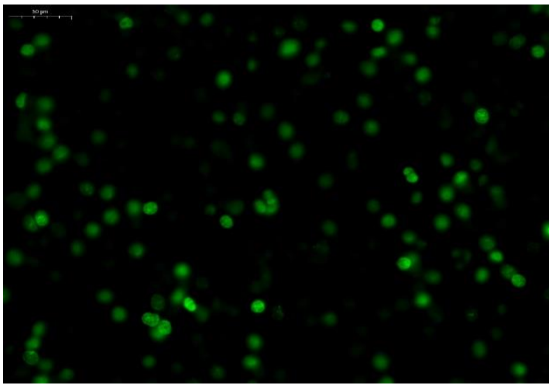

miR-542-3p mimics+ pcDNA3.1-ILK

Merge:

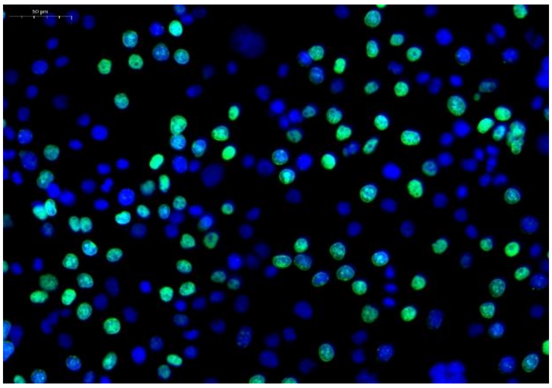

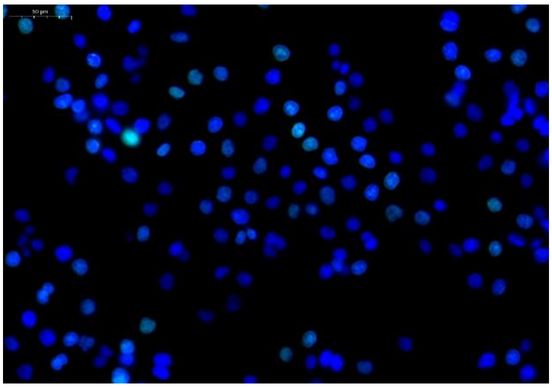

Blank 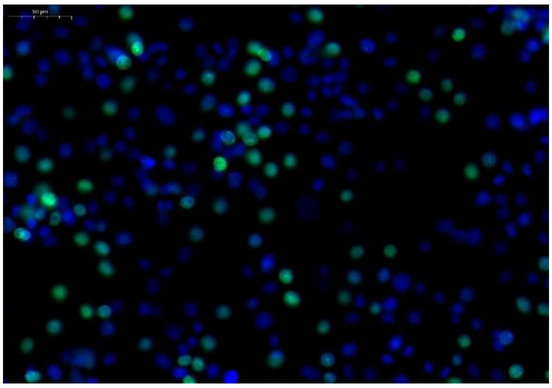 sh-CASC9-2 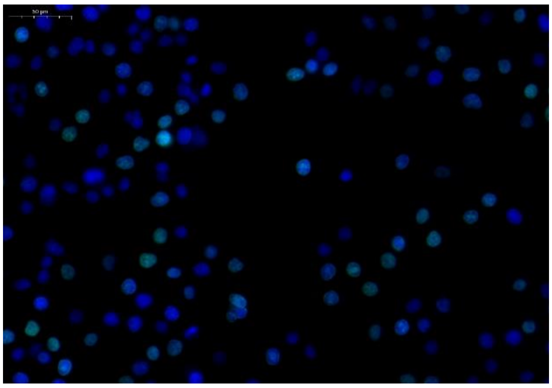

sh-CASC9-2+pcDNA3.1-ILK

miR-542-3p mimics

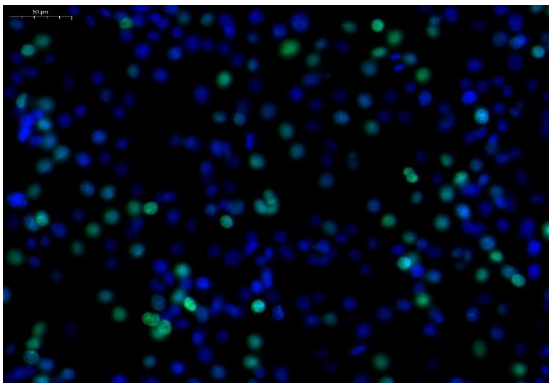

miR-542-3p mimics+ pcDNA3.1-ILK

2. Xenograft tumors (Fig3A)

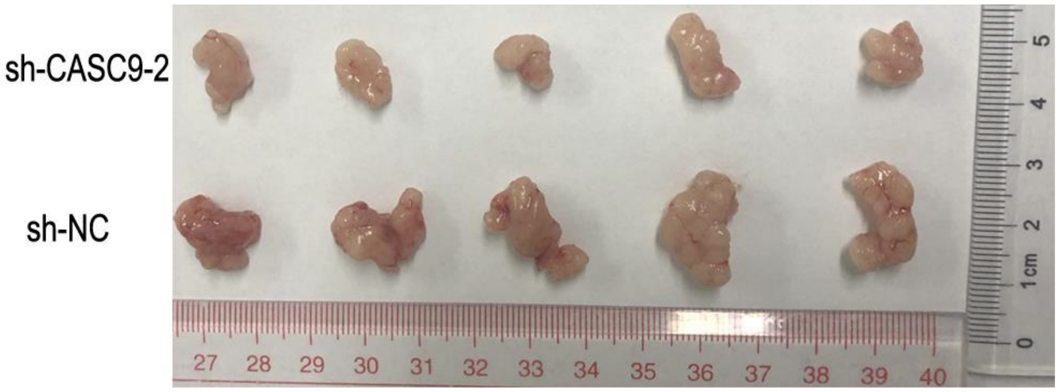

3. Transwell (Fig2E&F, Fig5E&F, Fig7A)

(1) Fig2E&F

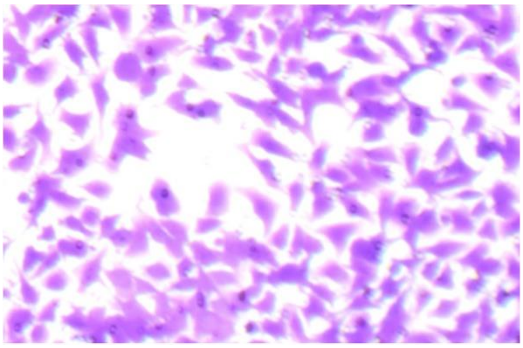

Fig2 SW620 Invasion sh-NC

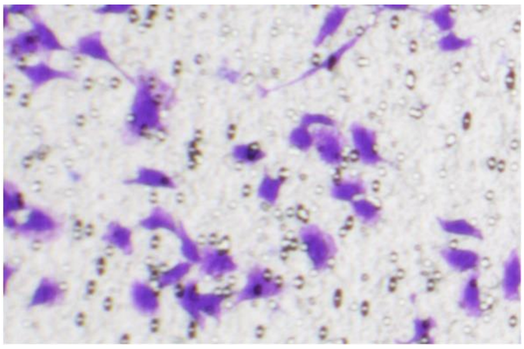

Fig2 SW620 Invasion sh-CASC9-2

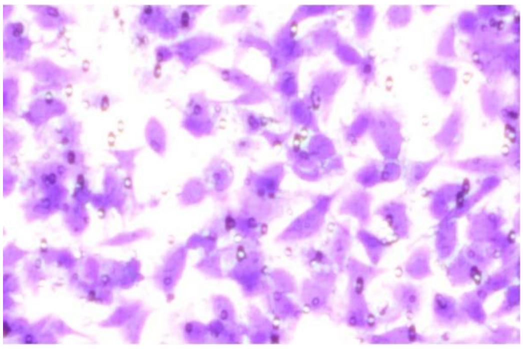

Fig2 HCT116 Invasion sh-NC

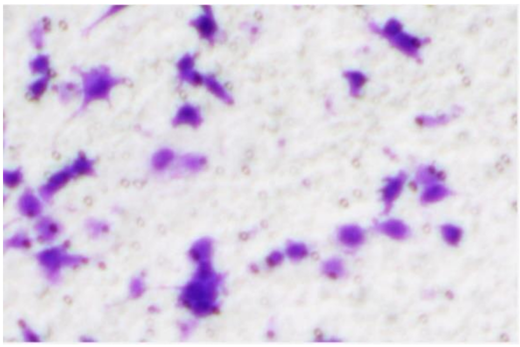

Fig2 HCT116 Invasion sh-CASC9-2

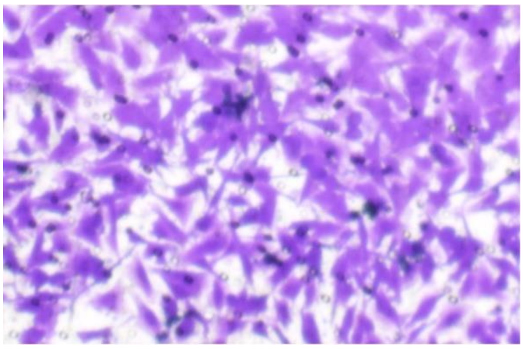

Fig2 SW620 Migration sh-NC

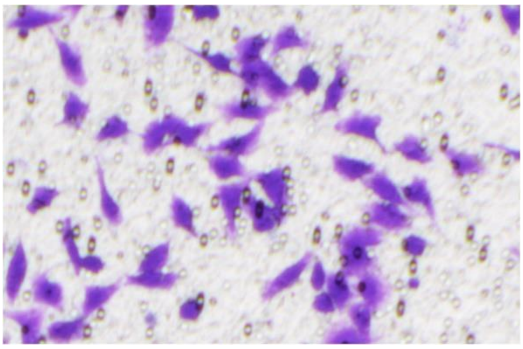

Fig2 SW620 Migration sh-CASC9-2

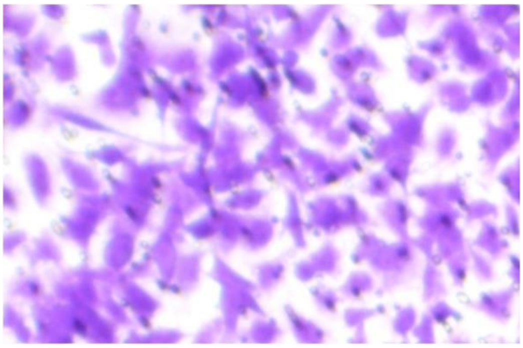

Fig2 HCT116 Migration sh-NC

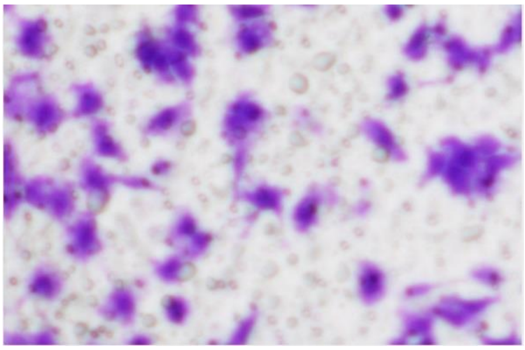

Fig2 HCT116 Migration sh-CASC9-2

(2) Fig5E&F

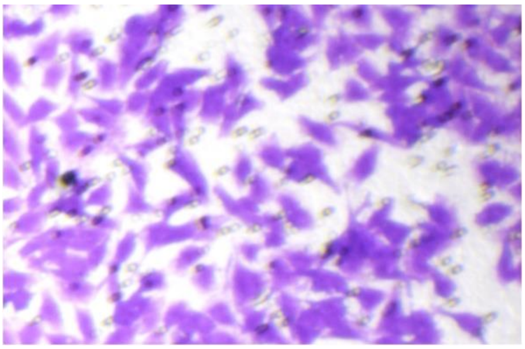

Fig5 SW620 Invasion miR-NC

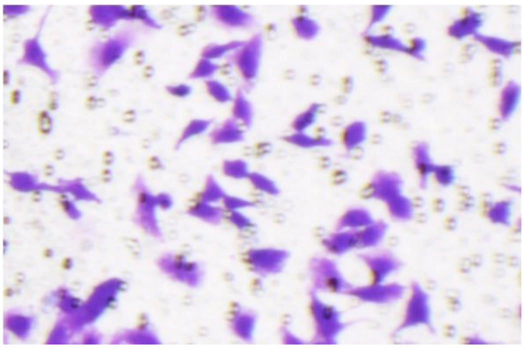

Fig5 SW620 Invasion miR-542-3p mimics

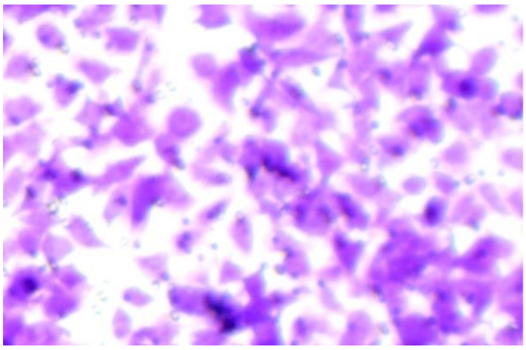

Fig5 HCT116 Invasion miR-NC

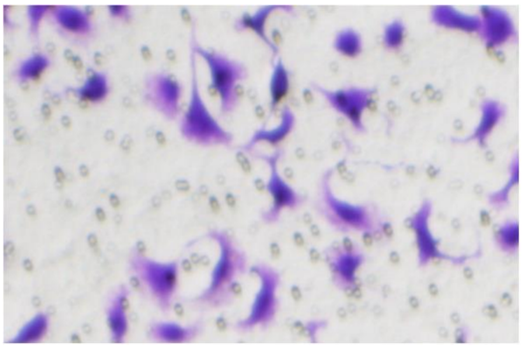

Fig5 HCT116 Invasion miR-542-3p mimics

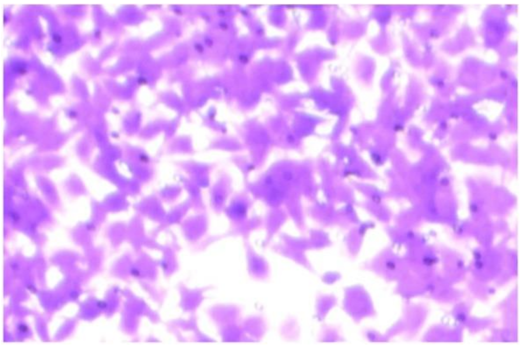

Fig5 SW620 Migration miR-NC

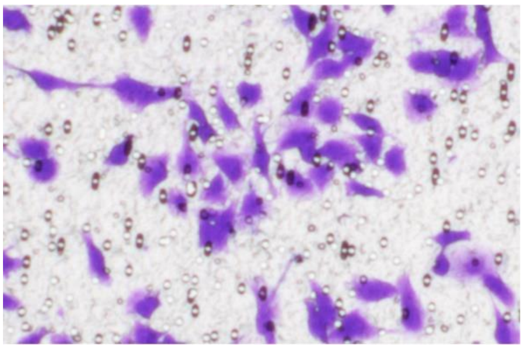

Fig5 SW620 Migration miR-542-3p mimics

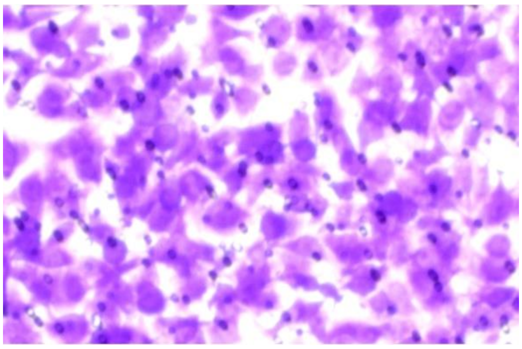

Fig5 HCT116 Migration miR-NC

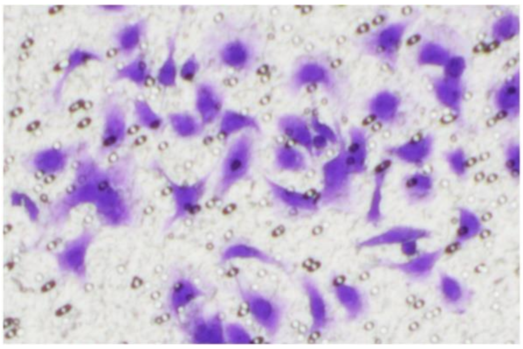

Fig5 HCT116 Migration miR-542-3p mimics

(3) Fig7A

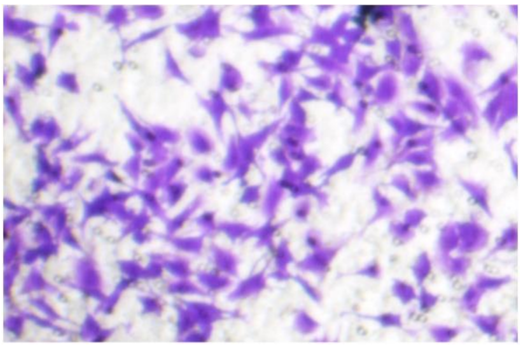

Fig7 SW620 Invasion Blank

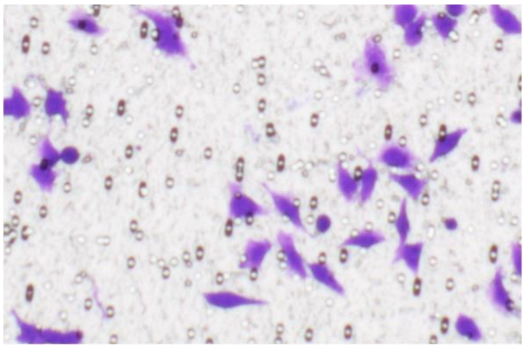

Fig7 SW620 Invasion sh-CASC9-2

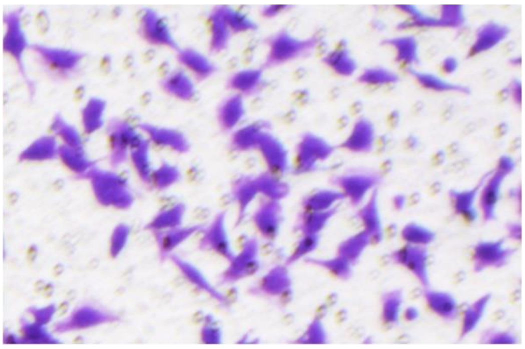

Fig7 SW620 Invasion sh-CASC9-2+pcDNA3.1-ILK

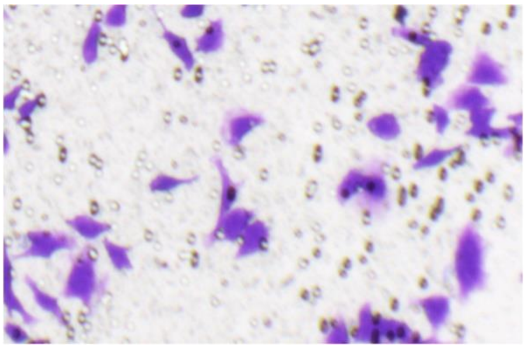

Fig7 SW620 Invasion miR-542-3p mimics

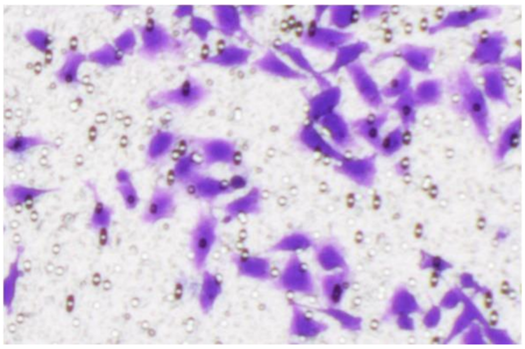

Fig7 SW620 Invasion miR-542-3p mimics+ pcDNA3.1-ILK

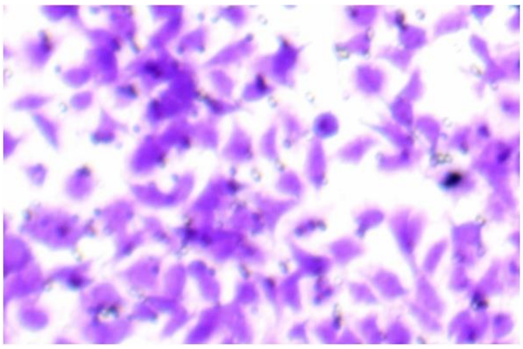

Fig7 HCT116 Invasion Blank

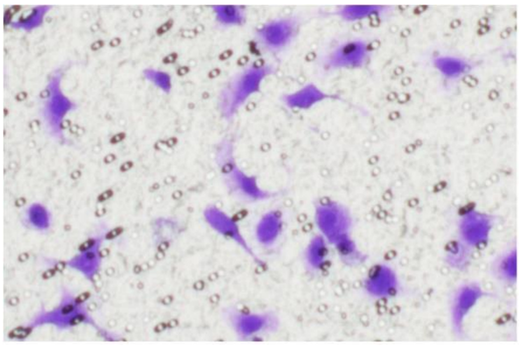

Fig7 HCT116 Invasion sh-CASC9-2

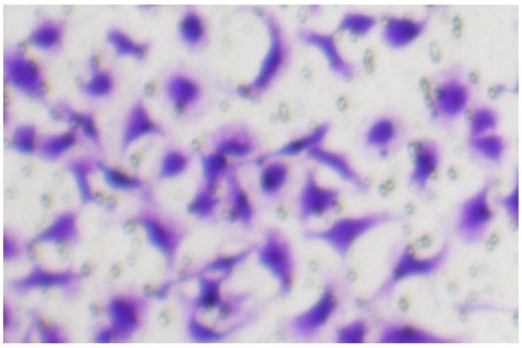

Fig7 HCT116 Invasion sh-CASC9-2+pcDNA3.1-ILK

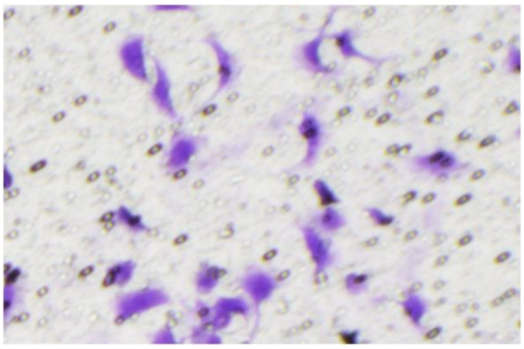

Fig7 HCT116 Invasion miR-542-3p mimics

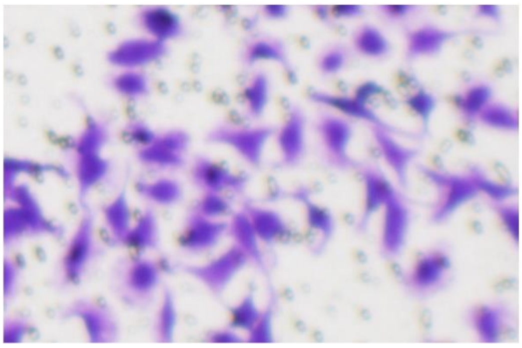

Fig7 HCT116 Invasion miR-542-3p mimics + pcDNA3.1-ILK

4. IHC (Fig3C&D)

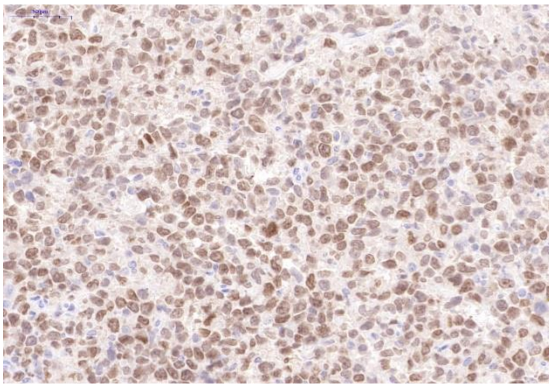

Ki67-40.0x-sh-NC

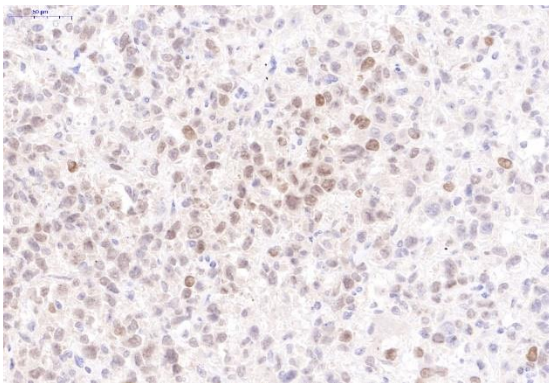

Ki67-40.0x-sh-CASC9-2

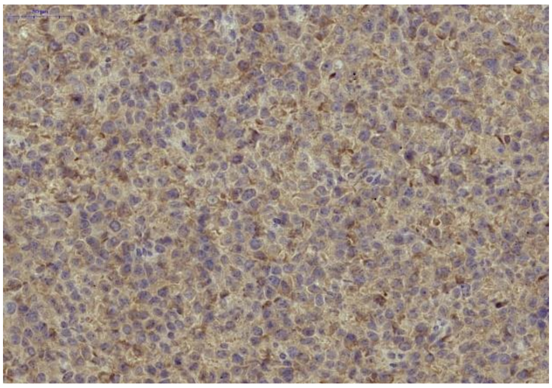

MMP2-40.0x-sh-NC

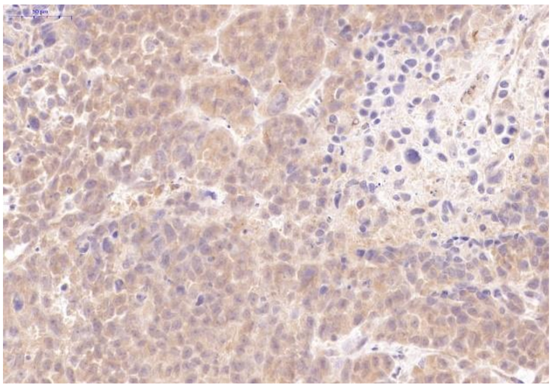

MMP2-40.0x-sh-CASC9-2

5. WB (Fig6C)

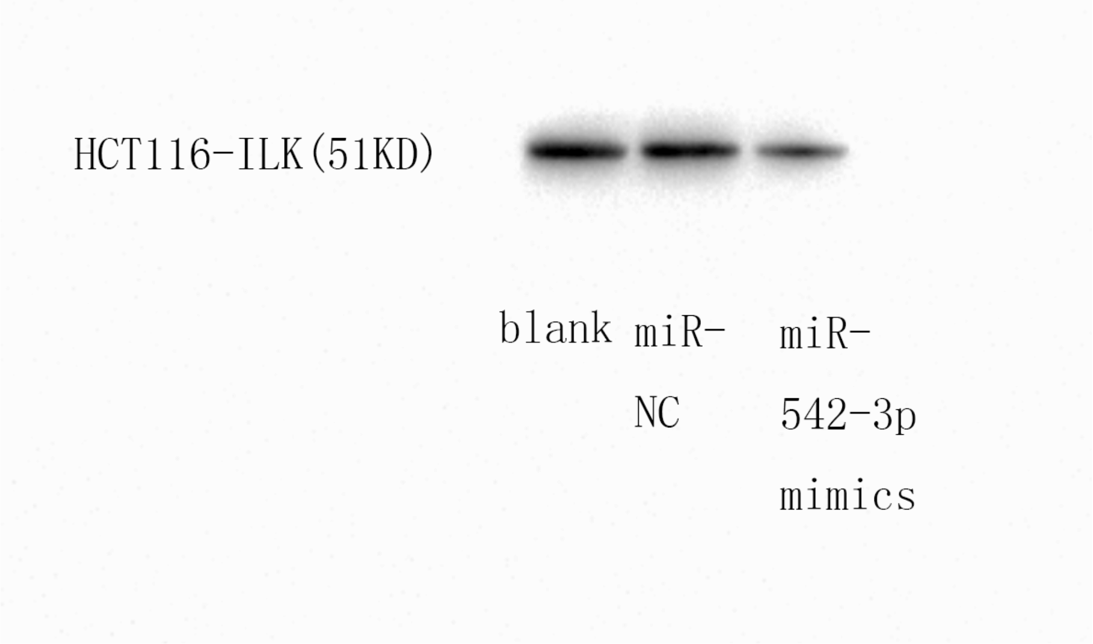
HCT116-ILK(51KD)
blank
miR-NC
miR-542-3p mimics

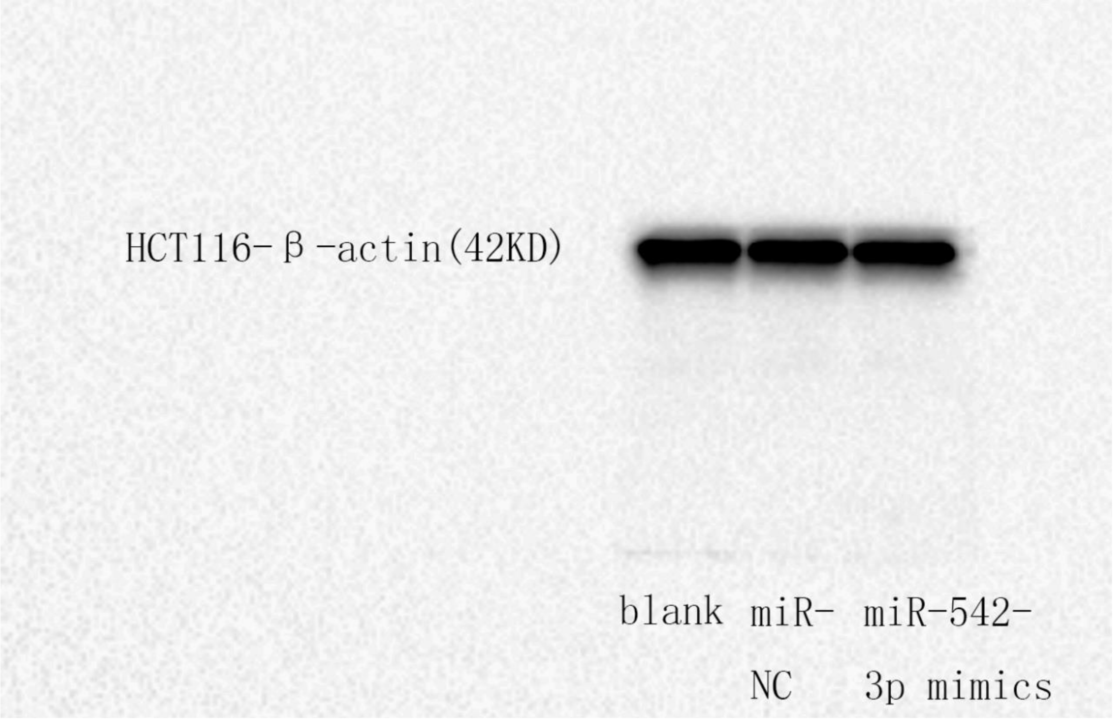
HCT116-β-actin(42KD)
blank
miR-NC
miR-542-3p mimics

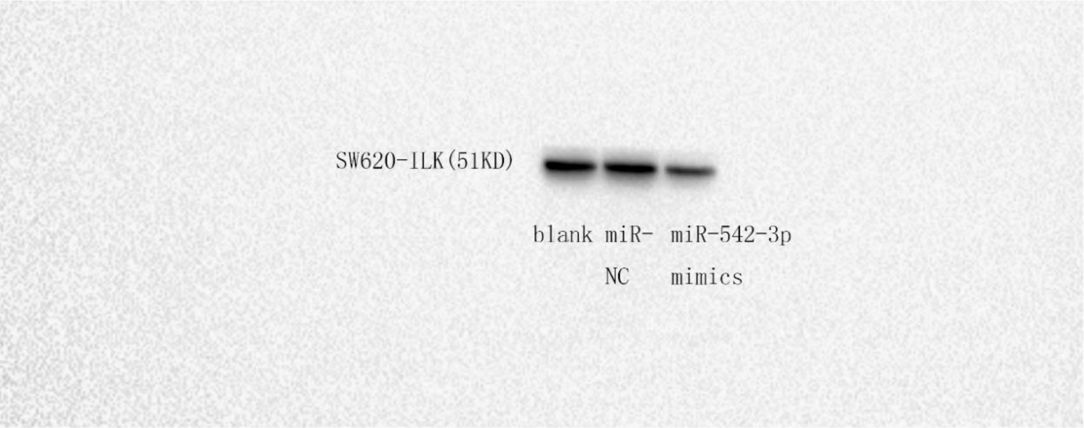
SW620-ILK(51KD)
blank
miR-NC
miR-542-3p mimics

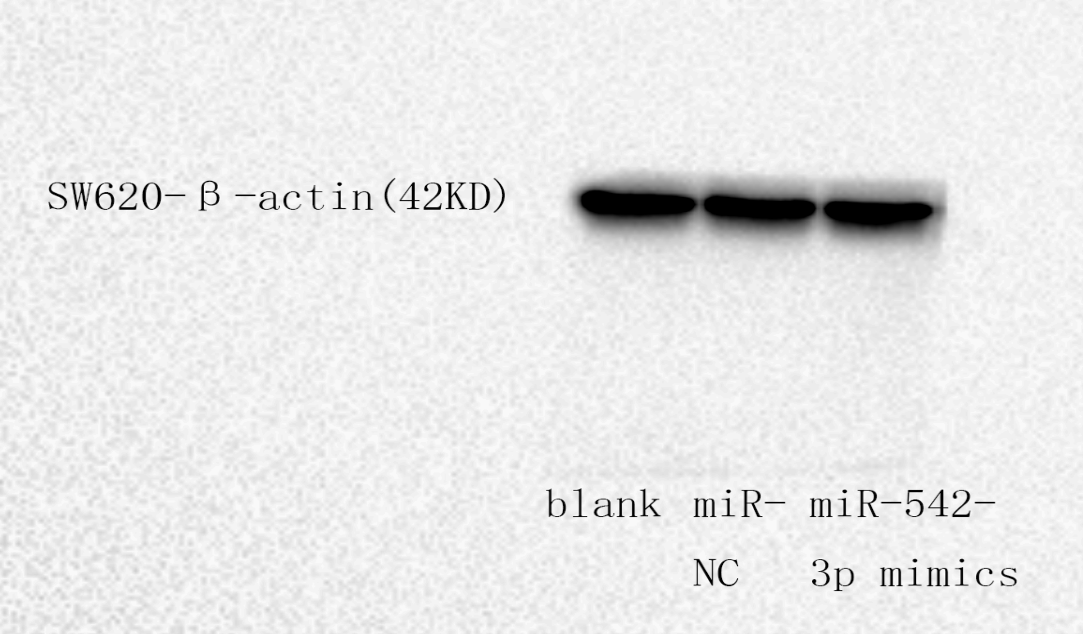
SW620-β-actin(42KD)
blank
miR-NC
miR-542-3p mimics
