# Supplementary material for: Silencing LncRNA CASC9 inhibits proliferation and invasion of colorectal cancer cells by MiR-542-3p/ILK
Source: PLoS One. 2022 Apr 15;17(4):e0265901. doi: 10.1371/journal.pone.0265901 (PMC9012350; doi:10.1371/journal.pone.0265901)
Supplement: S1 File — (PDF) [file pone.0265901.s002.pdf]

Fig1A
T/N
-3.4
-2.6
-2.2
-2.1
-1.6
-1.6
-0.8
-0.8
-0.7
-0.2
-0.1
0.0
0.3
0.4
0.5
0.8
1.0
1.2
1.2
1.3
1.3
1.5
1.9
2.1
2.4
2.4
2.7
2.8
3.2
3.4
3.6
3.7
3.7
4.4
4.7
5.6

Fig1B	
TNM I&II	TNM III
0.09	0.16
0.22	0.57
0.23	0.62
0.33	0.97
0.34	1.32
0.59	1.74
0.87	2.46
1.00	2.50
1.23	4.29
1.37	5.28
2.00	6.96
2.30	9.19
2.30	10.56
2.83	12.55
3.73	13.00
5.28	25.99
6.50	48.50
12.13	
21.11	

Fig1C		
NCM460	SW620	HCT116
1	18.94	17.53
0.92	22.31	15.23
1.08	17.68	19.41
0.93	23.18	17.62
1.07	19.03	17.66

Fig2A

	SW620			HCT116		
	Mean	SD	N	Mean	SD	N
sh-NC	1	0.05	5	1	0.06	5
sh-CASC9	0.72	0.04	5	0.64	0.04	5
sh-CASC9	0.32	0.02	5	0.36	0.02	5
sh-CASC9	0.57	0.03	5	0.71	0.05	5

Fig2B

	sh-NC			sh-CASC9-2		
	Mean	SD	N	Mean	SD	N
0h	0.25	0.03	3	0.25	0.03	3
24h	0.32	0.034	3	0.28	0.04	3
48h	0.62	0.07	3	0.41	0.045	3
72h	1.08	0.105	3	0.64	0.07	3

Fig2C

	sh-NC			sh-CASC9-2		
	Mean	SD	N	Mean	SD	N
0h	0.22	0.03	3	0.22	0.03	3

24h	0.3	0.034	3	0.29	0.04	3
48h	0.55	0.07	3	0.38	0.045	3
72h	1.01	0.105	3	0.63	0.07	3

Fig2D2

	SW620			HCT116		
	Mean	SD	N	Mean	SD	N
sh-NC	41.1	4.8	3	39.4	4.9	3
sh-CASC9	15.7	2.1	3	13.5	1.8	3

Fig2E2

	SW620			HCT116		
	Mean	SD	N	Mean	SD	N
sh-NC	223.5	26.2	3	167.2	19.6	3
sh-CASC9	47.2	6.8	3	44.3	6.7	3

Fig2F2

	SW620			HCT116		
	Mean	SD	N	Mean	SD	N
sh-NC	172.2	21	3	123	17.6	3
sh-CASC9	41	6.1	3	32	5.6	3

Fig3A2

	sh-NC			sh-CASC9-2		
	Mean	SD	N	Mean	SD	N
1w	162	20	5	75	13	5
2w	286	42	5	147	28	5
3w	527	102	5	220	62	5
4w	948	231	5	374	102	5

Fig3B		Fig3C2		Fig3D2	
sh-NC	sh-CASC9-2	sh-NC	sh-CASC9-2	sh-NC	sh-CASC9-2
878	346.1	105	42	105	72
972	192.1	108	30	107	60
669.2	334.5	92	41	93	76
1066.1	316.1	94	32	94	59
589.6	442.3	101	30	101	79

Fig3E		Fig3F		Fig3G	
sh-NC	sh-CASC9-2	sh-NC	sh-CASC9-2	miR-NC	sh-CASC9-2
1.0	0.3	0.8	3.5	1	0.45
0.9	0.4	1.0	4.0	1.01	0.37
1.1	0.3	1.0	3.5	1.17	0.45
1.0	0.2	1.1	3.1	0.95	0.4
1.0	0.3	1.1	3.4	0.87	0.58

Fig4B

	pmirGLO-CASC9-Wt			pmirGLO-CASC9-Mut		
	Mean	SD	N	Mean	SD	N
miR-NC	1	0.06	5	1.02	0.07	5
miR-542-3p	0.56	0.03	5	1.04	0.06	5

Fig4C

	IgG			Ago2		
	Mean	SD	N	Mean	SD	N
LncRNA C.	1	0.07	3	14.7	1.12	3
miR-542-3p	1	0.06	3	23.4	1.89	3

Fig4D

	IgG			Ago2		
	Mean	SD	N	Mean	SD	N
LncRNA C.	1	0.05	3	9.2	0.72	3
miR-542-3p	1	0.07	3	18.7	1.32	3

Fig4E

	SW620			HCT116		

	Mean	SD	N	Mean	SD	N
sh-NC	1	0.05	3	1	0.07	3
sh-CASC9	3.42	0.31	3	3.12	0.28	3

Fig5A

	SW620			HCT116		
	Mean	SD	N	Mean	SD	N
miR-NC	1	0.05	5	1	0.06	5
miR-542-3	8.3	0.52	5	7.8	0.47	5

Fig5B

	miR-NC			miR-542-3p mimics		
	Mean	SD	N	Mean	SD	N
0h	0.21	0.03	3	0.21	0.03	3
24h	0.28	0.034	3	0.27	0.04	3
48h	0.57	0.07	3	0.54	0.05	3
72h	1.14	0.12	3	0.82	0.085	3

Fig5C

	miR-NC			miR-542-3p mimics		
	Mean	SD	N	Mean	SD	N
0h	0.25	0.03	3	0.25	0.03	3
24h	0.38	0.034	3	0.32	0.04	3
48h	0.82	0.08	3	0.54	0.06	3
72h	1.09	0.11	3	0.67	0.085	3

Fig5D2

	SW620			HCT116		
	Mean	SD	N	Mean	SD	N
miR-NC	40.3	4	3	45	4.9	3
miR-542-3	16.7	1.8	3	14.7	1.6	3

Fig5E2

	SW620			HCT116		
	Mean	SD	N	Mean	SD	N
miR-NC	247	25.2	3	186.2	20.6	3
miR-542-3	58.2	7.8	3	52.3	7.4	3

Fig5F2

	SW620			HCT116		
	Mean	SD	N	Mean	SD	N
miR-NC	162.2	19.4	3	143	16.6	3
miR-542-3	45.3	6.6	3	39.2	5.2	3

Fig6B

	pmirGLO-ILK 3'UTR-Wt			pmirGLO-ILK 3'UTR-Mut		
	Mean	SD	N	Mean	SD	N
miR-NC	1	0.05	5	0.98	0.06	5
miR-542-3	0.52	0.04	5	1.02	0.06	5

Fig6C2

	SW620			HCT116		
	Mean	SD	N	Mean	SD	N
Blank	1	0.08	3	1	0.1	3
miR-NC	1.03	0.1	3	1.05	0.09	3
miR-542-3	0.46	0.04	3	0.39	0.04	3

Fig7A2

	SW620			HCT116		
	Mean	SD	N	Mean	SD	N
Blank	169.2	18.4	3	127.3	14.3	3
sh-CASC9	42.3	5.6	3	34.5	4.9	3
sh-CASC9	86.8	12.3	3	77.2	10.3	3
miR-542-3	50.3	7.2	3	36.1	5.1	3

miR542-3p	92.4	13.2	3	83.2	11.7	3

Fig7B2

	SW620			HCT116		
	Mean	SD	N	Mean	SD	N
Blank	43.2	3.4	3	47.6	3.9	3
sh-CASC9-	16.8	1.4	3	14.8	1.2	3
sh-CASC9-	31	2.7	3	28.3	2.3	3
miR-542-3p	17.5	1.4	3	14.4	1.4	3
miR542-3p	33.3	3	3	25.3	2.1	3
