# Supplementary figures and images for: Silencing LncRNA CASC9 inhibits proliferation and invasion of colorectal cancer cells by MiR-542-3p/ILK
Source: PLoS One. 2022 Apr 15;17(4):e0265901. doi: 10.1371/journal.pone.0265901 (PMC9012350; doi:10.1371/journal.pone.0265901)

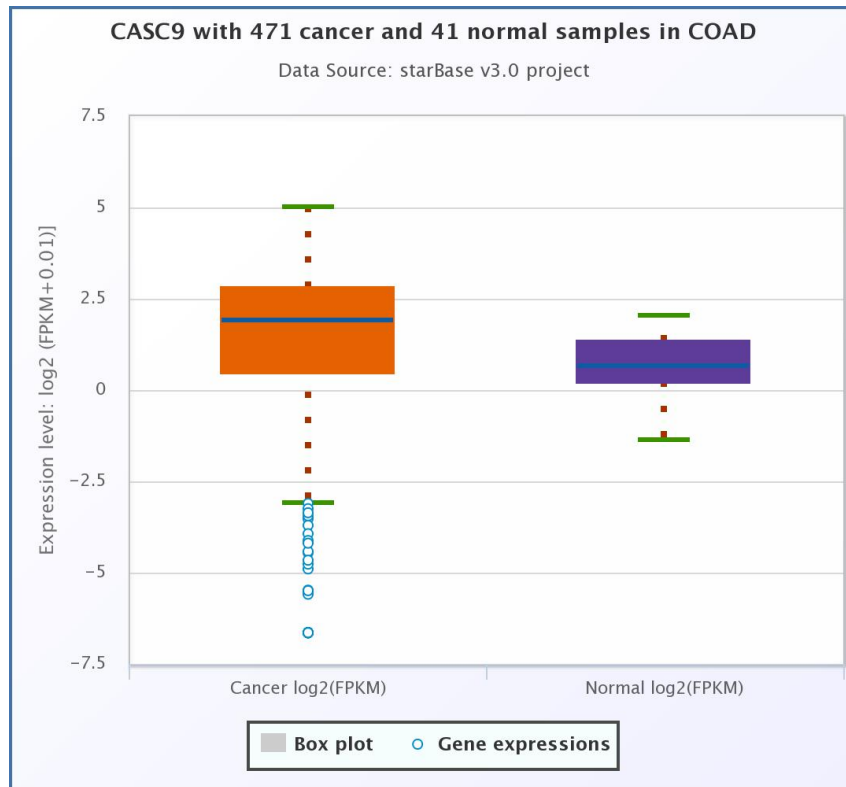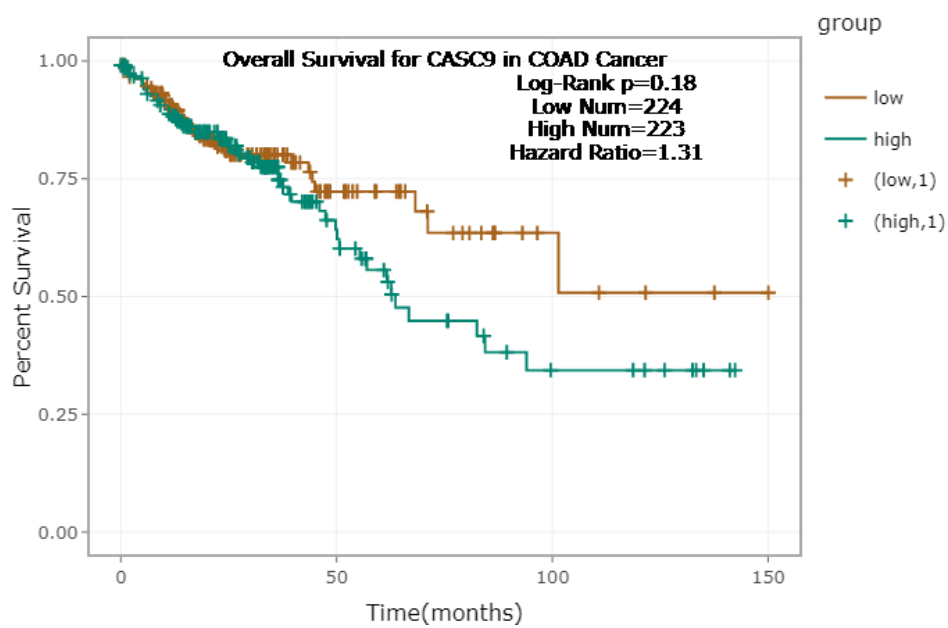

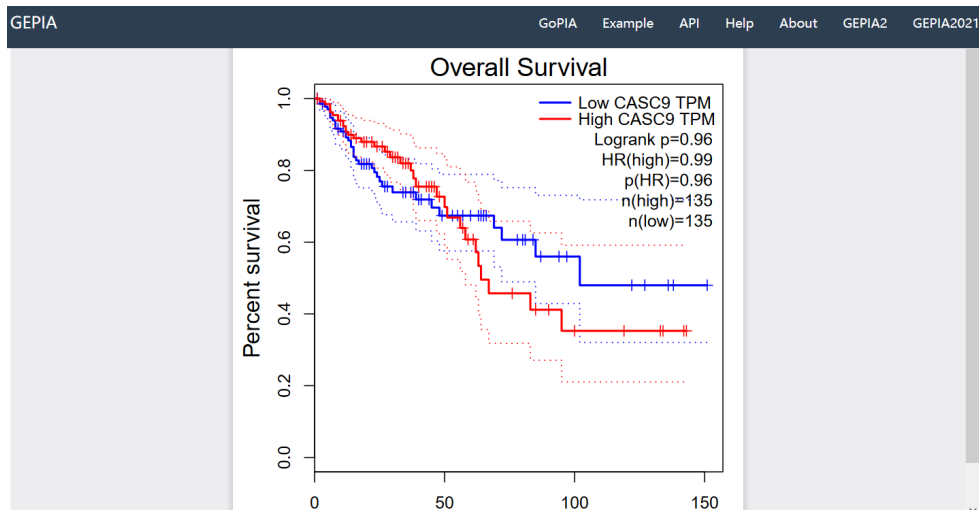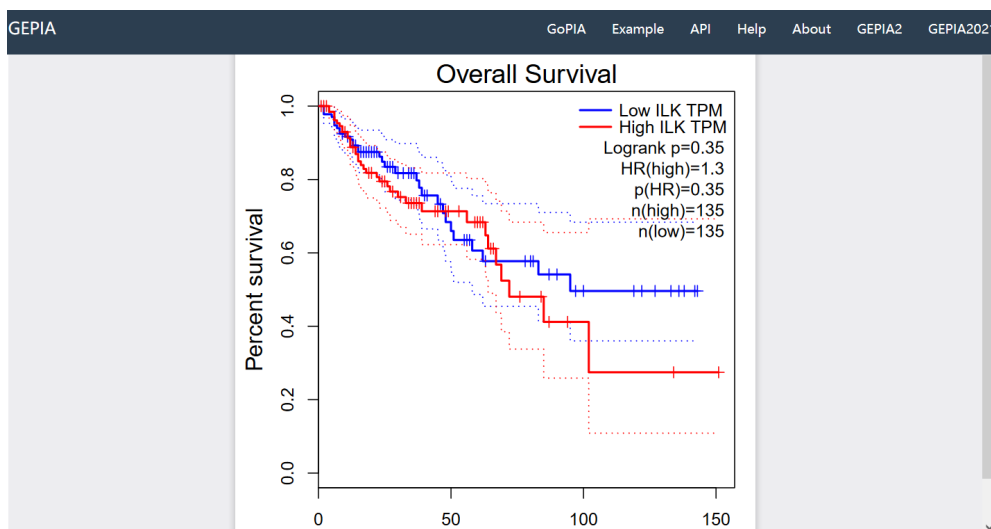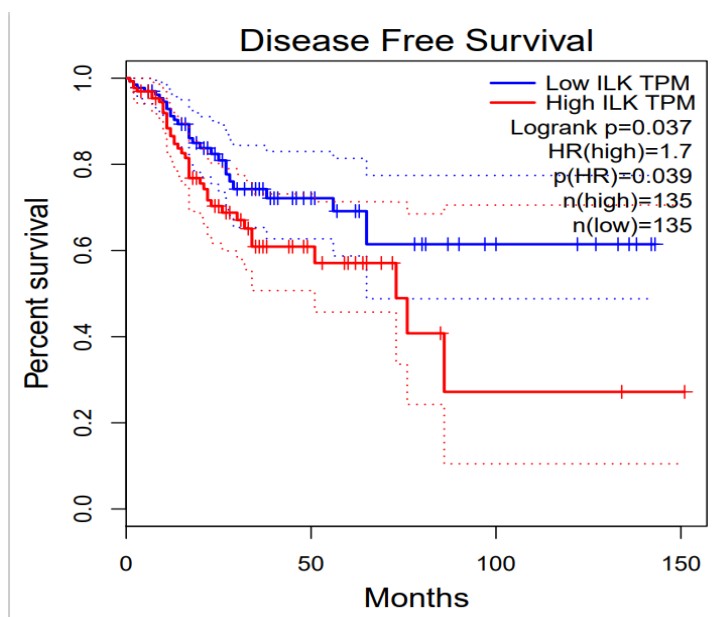

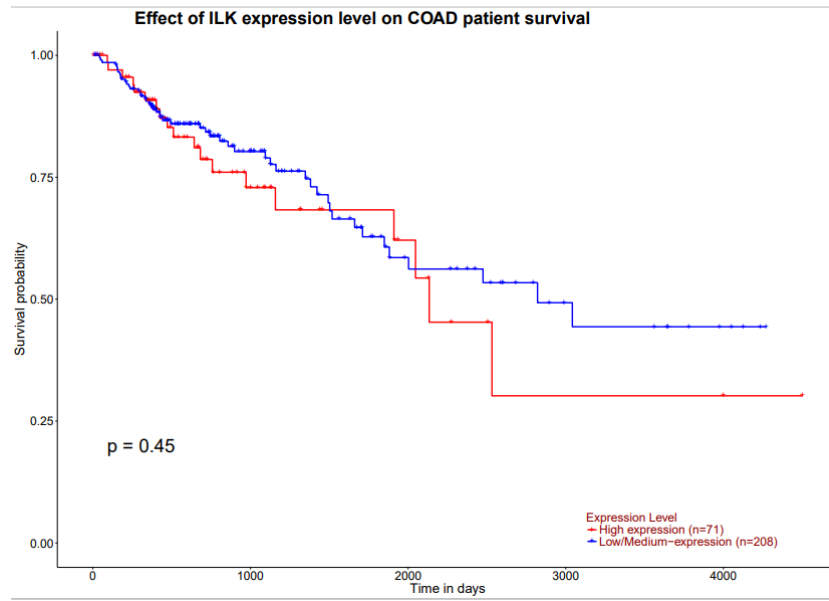

Supplement: S2 File — (PDF) [file pone.0265901.s003.pdf]
