# Supplementary material for: Silencing LncRNA CASC9 inhibits proliferation and invasion of colorectal cancer cells by MiR-542-3p/ILK
Source: PLoS One. 2022 Apr 15;17(4):e0265901. doi: 10.1371/journal.pone.0265901 (PMC9012350; doi:10.1371/journal.pone.0265901)
Supplement: Attachment — Submitted filename: Response to Reviewers.docx [file pone.0265901.s004.docx]

**Submission ID**: PONE-D-21-27936

**Title**: Silencing LncRNA *CASC9* Inhibits Proliferation and Invasion of Colorectal Cancer Cells by MiR-542-3p/ILK

**Journal**: PLOS ONE

**Response to Editor and Reviewers' comments**

Dear Emily Chenette and Reviewers,

We greatly appreciate this opportunity to resubmit a revised version of our manuscript named “Silencing LncRNA *CASC9* Inhibits Proliferation and Invasion of Colorectal Cancer Cells by MiR-542-3p/ILK” (manuscript ID: PONE-D-21-27936).

We are very pleased to address these comments of your esteemed reviewers, and we hope that our attention to these very constructive comments, are found to be responsive and received favorably by you and your expert reviewers.

Thank you for your consideration of our manuscript, and please do not hesitate to contact us if you have any further concerns.

Kind regards,

Name: Heping Zhang

Institutions: Department of anorectal, People's Hospital of Jiaozuo

Address: Jiefang Road No. 267, Shanyang District, Jiaozuo city, Henan Province, China

Email: zhanghepinglary@163.com

Editor’s comments:

Suggestion 1 for original research: Please revise the manuscript in response to all concerns of Reviewer 1.

**Response**: We appreciate the suggestion. The manuscript has been revised according to the concerns of Reviewer 1.

Suggestion 2 for original research: The manuscript is poorly written. Please have the whole manuscript edited well.

**Response:** We appreciate the suggestion. The manuscript has been polished by an English native speaker, and a certificate of embellishment was issued.

Suggestion 3 for original research: Please analyze the correlation of expression levels of lncCASC9, miR-542 and/or ILK in human tumor tissues using data base.

**Response:** We appreciate the suggestion. We used the database to directly analyze the correlation among lncCASC9, miR-542-3p and/or ILK, and found that there was a certain correlation between lncCASC9 and ILK in the survival trend, i.e., the higher the expression level, the shorter the survival period, but the p value had no statistical significance. It suggested that there may be some correlation between lncCASC9 and ILK. In addition, the correlation between miR-542-3p and lncCASC9 or ILK was not significant. Although there are certain data in TCGA database, due to the limitation of data, we have not analyzed the direct correlation among the three.

And the following contents have been added in discussion section in Page 17-18 Line 385-400: CASC9 is mainly concentrated in the cytoplasm and significantly upregulated in human tumor tissues including CRC. ILK, locating in the nucleus and centrosomes, is abnormally overexpressed in CRC tissues. We compared the survivals of CASC9 and ILK using GEPIA database (http://gepia.cancer-pku.cn/), and found that both CASC9 and ILK had the similar tendency that the survival time of the low gene expression group was longer than that of the high gene expression group over time, while the tendency in miR-542-3p was not obvious. It suggests that there may be some correlation between CASC9 and ILK. Some studies found that as a competing endogenous RNA, CASC9 inhibits miRNA expression by competitively binding to its target miRNA at the 3′UTR region via a sponging effect, and then regulates the progression of various human neoplasms. The expression of miR-542-3p in CRC tissues was really verified abnormally downregulated, which was verified in our study as well. What’s more, some researchers have demonstrated miR-542-3p overexpression down-regulating ILK then inhibiting osteosarcoma has been verified. Thus, there may be a potential mechanism that CASC9 negatively regulate miR-542-3p and miR-542-3p negatively regulate ILK in CRC, which was testified in our research.

Suggestion 4 for original research: Please analyze expression levels of LncCASC9, miR-542 and ILK in mouse tumor tissues.

**Response:** We appreciate the suggestion. The relevant analyzation has been added in the results section of Fig3 in Page 12-13 Line 260-264 & 277-280: The expression levels of lncRNA CASC9, miR-542-3p, and ILK on xenograft CRC were tested via qPCR. The results showed that lncRNA CASC9 and ILK were downregulated and miR-542-3p was upregulated (Fig 3E & 3F & 3G), which suggests that there may be a correlation among lncRNA CASC9, miR-542-3p and ILK.

Suggestion 5 for original research: MiR-542 has many targets as ILK. Please identify another target and show with ILK.

**Response:** We appreciate the suggestion. Of course, miR-542-3p has many other target genes which may participate in the biological behavior of CRC. For example, microR-542-3p mediates ILK and further inhibit cell proliferation, migration and invasion in osteosarcoma cells (DOI: 10.18632/aging.101698), miR-542-3p inhibits oral squamous cell carcinoma progression by inhibiting ILK/TGF-β1/Smad2/3 signaling (DOI: 10.18632/oncotarget.19986), miR-542-3p inhibits CRC cell proliferation, migration and invasion by targeting OTUB1 (PMCID: PMC5250690). The aim of our study is demonstrating that miR-542-3p/ILK exists in CRC based on our protocol, and our experiment proved that ILK is the target gene of miR-542-3p in CRC by rigorous logic. This pathway not always play a leading role, but it exists in CRC, and the existence of other pathways are not ruled out.

Please include the following items when submitting your revised manuscript:

A rebuttal letter that responds to each point raised by the academic editor and reviewer(s). You should upload this letter as a separate file labeled 'Response to Reviewers'.

A marked-up copy of your manuscript that highlights changes made to the original version. You should upload this as a separate file labeled 'Revised Manuscript with Track Changes'.

An unmarked version of your revised paper without tracked changes. You should upload this as a separate file labeled 'Manuscript'.

**Response:** We appreciate the suggestion. A rebuttal letter that responds to each point raised by the academic editor and reviewer 1 was uploaded as a separate file labeled 'Response to Reviewers'. A marked-up copy of our manuscript that highlights changes made to the original version was upload as a separate file labeled 'Revised Manuscript with Track Changes'. An unmarked version of our revised paper without tracked changes was upload as a separate file labeled 'Manuscript'.

Response to Reviewer 1：

Suggestion 1: Please check the English proficiency of the manuscript. There are some grammatical mistakes. For example, “We firstly detected the expression of lncRNA CASC9, then evaluated the effect of lncRNA CASC9 on CRC, and how miR-542-3p/ILK to affect lncRNA CASC9.” in the study design would be better changed to “and explored how miR-542-3p/ILK affects lncRNA CASC9.”

**Response 1：**We apologize for the grammatical mistakes. We have corrected it to “We firstly detected the expression of lncRNA CASC9, then evaluated the effect of lncRNA CASC9 on CRC, and explored how miR-542-3p/ILK to affect lncRNA CASC9” in the revised manuscript in Page 4 Line 83.

Suggestion 2. There should be a space between the number and the unit. For example, it should be “8 μg/ml”, “0.5 μg/ml”, “1 h” and “30 μg” but not “8μg/ml”, “0.5μg/ml”, “1h” and “30μg”.

**Response 2：**We appreciate the suggestion. A space between the number and the unit has been added in the revised manuscript.

Suggestion 3. It is recommended that the method and the test are stated in full in the subtitle, e.g., "EdU assay for cell proliferation".

**Response 3：**In fact, both abbreviations and detailed names of method and the test can be seen in not a few papers (e.g., https://doi.org/10.1371/journal.pone.0256995, https://doi.org/10.1371/journal.pone.0258061, https://doi.org/10.1371/journal.pone.02 53877, etc.). For ease of understanding, we have detailed them according to your suggestion as possible.

Suggestion 4. The reference should be provided for “IHC assay was performed as described previously using supersensitive horseradish peroxidase immunohistochemistry kit (Sangon Biotech, Shanghai, China) per manufacturer’s instructions……” in “Immunohistochemistry”.

**Response ：**We appreciate the suggestion. The reference had been provided for “IHC assay was performed as described previously using supersensitive horseradish peroxidase immunohistochemistry kit (Sangon Biotech, Shanghai, China) [3] per manufacturer’s instructions……” in “Immunohistochemistry” in the revised manuscript in Page 8 Line 168.

Suggestion 5. For the p value, please unify the case of “p” in the statistical analysis and result.

**Response ：**We apologize for the editing mistake. P value has been unified in the statistical analysis and results in the revised manuscript.

Suggestion 6. For Fig.4b and Fig.6b, “Dual luciferase report assay revealed that compared to 3 control subsets, the activity in Mut significantly reduced” did not match with the data shown in the figures. Please check them.

**Response ：**We apologize for the editing misunderstanding. There needs to be some explanation here that we changed the letters from lowercase to uppercase in figures and manuscript, as Fig4b to Fig4B. We checked Fig.4 and Fig.6 again, and thought the original description were less rigorous. We have made modifications for the results and annotations of Fig.4B and Fig.6B in the revised manuscript in Page 13 Line 284-288 & 297-299 and Page 15-16 Line 339-342 & 348-350.

In Fig.4B, the original sentence has been corrected into “dual-luciferase reporter gene assay with lncRNA CASC9 plasmid vector including Wt and Mut were designed. The results showed that the luciferase activity of the Wt vector in the lncRNA CASC9-Wt+miR-542-3p mimics subset was significantly reduced compared with lncRNA CASC9-Wt+NC subset, but had no effect on that of Mut vector (Fig 4B)”.

In Fig.6B, original sentence has been corrected to “Dual-luciferase reporter gene assay with ILK plasmid vector including Wt and Mut 3’ UTR were designed. The results showed that the luciferase activity of the Wt reporter in the ILK-Wt+miR-542-3p mimics subset was significantly reduced compared with ILK-Wt+NC subset, while had no effect on that of Mut vector (Fig 6B).”

Suggestion 7. There should be a scale bar to show the size of the cells for EdU and transwell assay in figures.

**Response 7：**We appreciate the suggestion. Scale bars on images of EdU in Fig2D & Fig5D & Fig7B and images of immunohistochemistry in Fig3C&D in original manuscript were exist, which are unclear after resizing images, and were enhanced in the revised manuscript. In addition, we checked the original images of transwell assay in Fig2E&F, Fig5E&F and Fig7A, and added the scale bars in the revised manuscript.
